# Do preoperative enlarged lymph nodes affect the oncologic outcome of laparoscopic radical gastrectomy for gastric cancer?

**DOI:** 10.18632/oncotarget.12549

**Published:** 2016-10-09

**Authors:** Qi-Yue Chen, Chang-Ming Huang, Chao-Hui Zheng, Ping Li, Jian-Wei Xie, Jia-Bin Wang, Jian-Xian Lin, Jun Lu, Long-Long Cao, Mi Lin, Ru-Hong Tu

**Affiliations:** ^1^ Department of Gastric Surgery, Fujian Medical University Union Hospital, Fuzhou, Fujian, China

**Keywords:** gastric carcinoma, enlarged LN, lymphadenectomy, surgical outcomes, propensity score matching

## Abstract

**Background:**

To investigate the long-term outcome of laparoscopic radical gastrectomy (LAG) for gastric cancer (GC) with preoperative enlarged lymph nodes (LNs).

**Materials and Methods:**

We retrospectively collected data on 855 patients who underwent LAG for GC. The patients were divided into large (>10 mm) and small (=10 mm) LN groups (LG and SG) based on the preoperative size of the LNs. The outcomes were compared using a 1:1 propensity score-matching method. The enlarged LNs were divided into five areas according to their location.

**Results:**

Before matching, the LG was associated with more retrieved LNs than the SG, whereas after matching, the numbers of LNs retrieved were similar. These numbers remained similar as the number of areas with enlarged LNs increased. Before matching, patients in the LG demonstrated a significantly lower 3-year overall survival rate than those in the SG (*p* < 0.001). Additionally, in the LG, 3-year overall survival rates were similar among patients with different total numbers of areas with enlarged LNs. After matching, the 3-year overall survival rate of the LG was close to that of the SG (81.1% *vs*. 72.4%, *p* = 0.066). A stratified analysis according to the only independent prognostic factor (pTNM stage) demonstrated that the 3-year overall survival rates at each stage were similar between the LG and SG.

**Conclusions:**

LAG has similar oncologic outcomes for GC with or without preoperative enlarged LNs in the same tumor stage. Furthermore, the total number of areas with enlarged LNs has no impact on the long-term outcome.

## INTRODUCTION

Lymph node (LN) metastasis (LNM) is the most common pattern of metastatic spread in gastric cancer (GC) [1–3]. Therefore, GC, especially in advanced stages, presents with enlarged LNs. Only by complete dissection of perigastric LNs, including enlarged LNs, can an ideal curative operation for GC be achieved. Since the first reported laparoscopic gastrectomy for early GC was described by Kitano in 1994 [4], this technical approach has been adopted because its minimally invasive nature confers advantages over open surgery, such as less blood loss, reduced pain, accelerated recovery and better cosmetic results. Laparoscopic gastrectomy provides not only favorable short-term outcomes but also equivalent oncologic outcomes [5–7]. Improvements in laparoscopic techniques and surgical instruments have led to the development of laparoscopic-assisted gastrectomy with lymphadenectomy for GC. Accordingly, laparoscopic radical gastrectomy (LAG) has been increasingly performed for advanced GC, with good LN dissection efficacy and outcomes, as reported by several retrospective studies [8–10].

However, enlarged LNs increase the difficulty of lymphadenectomies for GC, although whether preoperative enlarged LNs affect the radical and long-term oncologic results of LAG for GC with enlarged LNs has yet to be determined. The purpose of this study was thus to compare the long-term oncologic outcomes of LAG for GC with and without preoperative enlarged LNs using a propensity score-matching method.

## MATERIALS AND METHODS

### Patients

From December 2009 to May 2012, 904 patients with GC who had undergone LAG performed by the same group of surgeons at Fujian Medical University Union Hospital in China were identified in a retrospectively maintained database. Patients with T4b (*n* = 40), remnant GC (*n* = 8) and splenectomy due to enlarged LNs at the splenic hilus (*n* = 1) were excluded. Therefore, in total, 855 patients were included in this study. The diameter of each LN was measured using transverse multidetector-row computed tomography (MDCT) images and was recorded by two experienced radiologists. LNs with a long-axis diameter greater than 10 mm were regarded as clinically metastasized according to prior studies [11–14]. Based on the preoperatively measured long-axis diameter of their LNs, the patients were divided into large ( > 10 mm) and small (≤10 mm) LN groups (LG and SG). In all, 571 patients were included in the LG, and 284 patients were in the SG.

The propensity scores were calculated using a logistic regression model and the following covariates: age, sex, BMI, Charlson score, tumor location, clinical TNM (cTNM) stage, tumor size, resection method and extent of lymphadenectomy. We employed a computerized technique to match the closest available scores without replacement using SPSS 18.0 (SPSS Inc., Chicago, IL, USA). A total of 218 patients in each group (LG and SG) were examined, and we compared the long-term oncologic outcomes between the two groups. Written informed consent was obtained from all patients prior to their operations. This study was approved by the institutional review board of Fujian Medical University Union Hospital (approval number: 20070428).

Preoperative imaging studies were routinely performed following endoscopic examination and upper gastrointestinal contrast to confirm the tumor location. These studies included chest radiography, computed tomography (CT) scanning, and ultrasonography (US) of the abdomen as well as bone scanning and positron emission tomography-CT (PET-CT) as needed to evaluate the diameter of the LNs and the existence of distant metastasis. According to a prior study [15], we considered an LN as positive if the longest diameter was > 1.0 cm or if it was 0.7-1.0 cm and showed strong enhancement, a round shape, central necrosis, or perinodal infiltration, all of which suggest metastasis. We used CT scans and the 7th edition of the Union for International Cancer Control (UICC) classification system to assess the clinical stage [16].

The inclusion criteria were as follows: histologically confirmed GC based on analyses of endoscopic biopsy specimens, GC pathologically confirmed as stage T1-T4a, no evidence of distant metastasis based on the aforementioned examinations, no evidence of invasion of adjacent organs (such as the pancreas, spleen, liver, or colon) or obvious enlarged LNs around the abdominal aorta, and completion of curative R0 resection according to the pathological diagnosis after surgery. The exclusion criteria included the following: remnant GC, intraoperative evidence of peritoneally disseminated or distant metastasis, GC pathologically confirmed as stage T4b or combined major organ resection, incomplete pathological data, combined splenectomy due to enlarged splenic hilar LNs, and conversion to open surgery.

### Variable definitions

It is difficult to accurately confirm to which nodal group enlarged LNs belong before operation. Thus, in the present study, the enlarged LNs were divided into five areas according to their approximate anatomical location. Specifically, No. 14v and No. 6 LNs were considered to be in area I; No. 7, No. 8a, No. 9 and No. 11p LNs, in area II; No. 12a and No. 5 LNs, in area III; No. 1 and No. 3 LNs, in area IV; and No. 4 LNs, in area V.

### Surgical procedures

The sequence of the lymphadenectomy was as follows: (1) For distal gastrectomy: No. 6 → No. 7, 9, 11p → No. 3, 1 → No. 8a, 12a, 5 → No. 4sb. (2) For total gastrectomy: No. 6 → No. 7, 9, 11p → No. 8a, 12a, 5 → No. 1 → No. 4sb → No. 10, 11d → No. 2. For details, please see these references [17–20].

### Follow-up

Postoperative follow-ups were performed every 3 months for 2 years and then every 6 months from years 3-5. Most routine patient follow-up appointments included a physical examination, laboratory tests (including assessment of CA19-9, CA72-4, and CEA levels), chest radiography, abdominopelvic US or CT and an annual endoscopic examination. Overall survival was calculated from the day of surgery until death or until the final follow-up date, whichever occurred first.

### Statistical analysis

All of the statistical analyses were performed using SPSS 18.0 for Windows (SPSS Inc., Chicago, IL, USA). All continuous variables were presented as the median (range). McNemar's test or Fisher's exact tests were used for categorical variables with more than 2 levels, accounting for the matched design of the propensity score-matched sample. Cumulative survival rates were compared using the Kaplan-Meier method and log-rank test. Regression analysis was performed using the Cox proportional hazards regression model in multivariate analyses. Values of *p* < 0.05 were considered statistically significant.

## RESULTS

### Incidence of enlarged LNs and the distribution of their areas

A total of 66.8% (571/855) of the patients had enlarged LNs, and the incidences of enlarged LNs in areas I, II, III, IV and V were 23.9% (204/855), 51.3% (439/855), 9.8% (84/855), 46.9% (401/855) and 12.7% (109/855), respectively. The frequencies of enlarged LNs in patients with different total numbers (1, 2, 3, 4 or 5) of areas with enlarged LNs were 18.2% (156/855), 26.3% (225/855), 15.9% (136/855), 5.4% (46/855) and 0.9% (8/855), respectively.

### Patient characteristics before and after matching

Table 1 shows the clinicopathologic characteristics of all of the patients (*n* = 855) and the propensity score-matched patients in particular (*n* = 436). Before matching, BMI, Charlson score, extent of lymphadenectomy, cTNM stage, use of neoadjuvant chemotherapy (NC) and adjuvant chemotherapy, operation time, reconstruction method, gastrectomy extent, tumor size, median diameter of the LNs, pT stage, pN stage and pTNM stage significantly differed between the LG and SG. After matching, only pN stage, median diameter of the LNs and use of NC significantly differed between the two groups.

**Table 1:** Patients’ Clinicopathological Characteristics Before and After Matching

Characteristic	All Patients		*p*	Propensity-Matched Patients				*p*
	SG (*n* = 284)	LG (*n* = 571)		SG (*n* = 218)		LG (*n* = 218)		
Age (years)			0.457					0.921
<65	169	355		135		137		0.824
≥65	115	216		83		81		
Gender			0.932					0.82
Male	217	439		169		166		0.866
Female	67	132		49		52		
BMI (kg/m^2^)^a^			<0.001					0.793
<25	219	504		182		185		
≥25	65	67		36		33		
ASA			0.439					0.883
I	95	213		80		85		
II	177	340		132		127		
III	12	18		6		6		
Previous abdominal surgery surgery			0.157					0.217
No	241	505		182		192		
Yes	43	66		36		26		
Charlson score			0.049					0.235
0	188	407		151		158		
1-2	88	159		62		59		
≥3	8	5		5		1		
Lymphadenectomy			<0.001					0.451
D1+	106	79		55		63		
D2	178	492		163		155		
Neoadjuvant			<0.001					<0.001
chemotherapy								
Yes	2	17		1		6		
No	282	554		217		212		
Reconstruction			<0.001					0.919
Roux-en-Y	114	311		96		89		
B-I	143	226		105		110		
B-II	11	20		6		7		
Esophagogastric	16	14		11		12		
Operative time (mins)	163.9±40.0	174.5±50.7	0.002 .0	163.8±38.0		166.6±51.8		0.521
Intraoperative blood loss (ml)	62.5±81.2	82.6±165.5	0.05	65.4±91.8		69.1±99.7		0.961
Gastrectomy extent			<0.001					0.792
Total	114	311		96		89		
Distal	154	246		111		117		
Proximal	16	14		11		12		
Tumor location			0.032					0.711
Upper	66	139		53		43		
Middle	46	113		38		40		
Lower	150	248		109		117		
≥2 areas	22	71		18		18		
cTNM stage			<0.001					0.193
IA	109	87		61		57		
IB	36	40		30		22		
IIA	25	66		26		26		
IIB	31	59		21		23		
IIIA	26	61		27		15		
IIIB	24	94		13		19		
IIIC	33	164		40		56		
Tumor diameter (cm)			<0.001					0.942
<2	106	95		66		66		
2-5	103	156		81		78		
≥5	75	320		71		74		
Median LN			<0.001					<0.0011
diameter (cm)								
	0.4(0-1.0)	1.5(1.1-5.8)		0.5(0-1.0)		1.5(1.1-5.8)		
Histologic type			0.744					0.824
Differentiated	75	158		55		52		
Undifferentiated	209	413		163		166		
Adjuvant			0.504					0.879
chemotherapy								
Yes	185	385		140		148		
No	99	186		78		70		
pT stage			<0.001					0.702
T1	120	111		68		76		
T2	35	58		32		25		
T3	69	182		61		58		
T4a	60	220		57		59		
pN stage			<0.001					0.019
N0	162	172		106		98		
N1	44	83		41		30		
N2	34	86		30		22		
N3	44	230		41		68		
pTNM stage			<0.001					0.08
IA	109	87		58		66		
IB	36	40		33		18		
IIA	25	66		22		25		
IIB	31	59		29		19		
IIIA	26	61		22		18		
IIIB	24	94		23		24		
IIIC	33	164		31		48		

ASA: American Society of Anesthesiologists

### Surgical quality of LN dissection

Before matching, more LNs were retrieved in the LG than in the SG, and the number of LNs retrieved significantly differed with the total number of areas with enlarged LNs (*p* = 0.004). Stratified analysis also showed that patients in the LG had significantly more LN dissections than patients in the SG when in the pN1 stage, whereas comparable numbers of retrieved LNs were found for patients in the pN0, pN2 or pN3 stage between the two groups.

**Figure 1:**
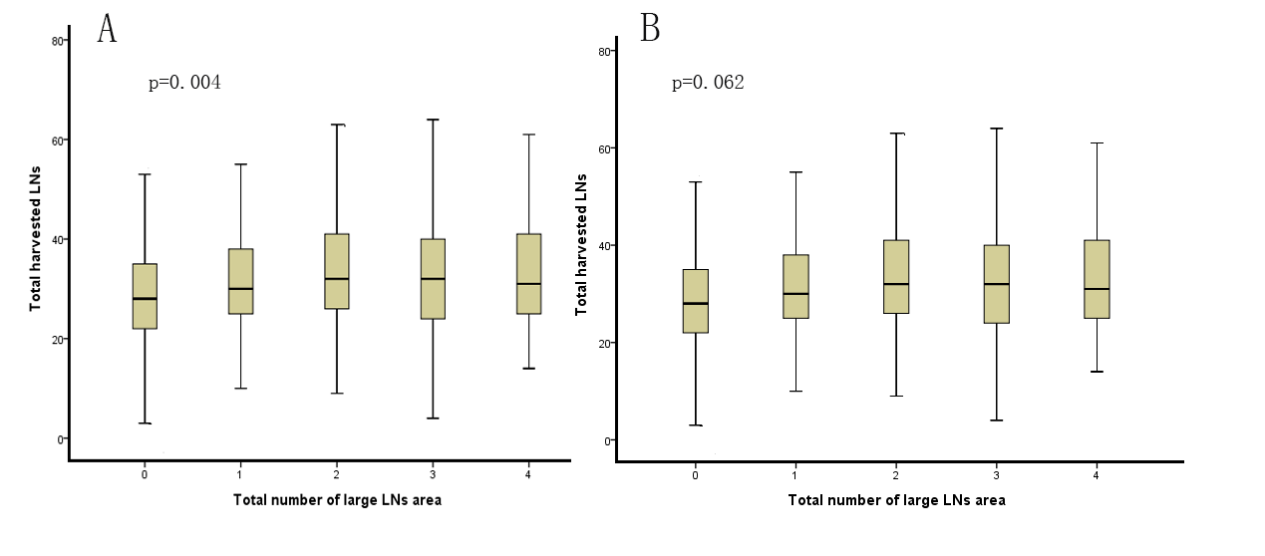
Comparison of the total number of areas with enlarged lymph nodes and the mean number of harvested lymph nodes before and after matching

After matching, although LNM was significantly more frequent in the LG compared with the SG, the total numbers of retrieved LNs in the stratified analysis according to pN stage were similar between the two groups and remained similar as the number of areas with enlarged LNs increased (*p* = 0.062) (Table 2 and Figure 1).

**Table 2:** Lymph Node Dissection Before and After Matching

Characteristic	All Patients		*p*	Propensity-Matched Patients			*p*
	SG (*n* = 284)	LG (*n* = 571)		SG (*n* = 218)	LG (*n* = 218)	
Harvested LNs	29.6±11.2	32.6±12.0	0.001	30.2±11.4		31.9±12.0		0.116
pNo	28.6±11.1	29.6±12.4	0.474	29.5±12.0		29.1±12.3		0.824
pN1	28.0±11.2	32.5±11.6	0.04	28.5±11.3		33.5±11.2		0.069
pN2	31.2±8.6	32.2±11.4	0.633	30.5±8.5		31.3±9.4		0.745
pN3	33.8±12.4	35.0±11.6	0.554	33.3±11.7		35.5±11.9		0.347
Areas with enlarged LNs			0.004					0.062
0	29.6±11.2			30.2±11.4				
1	31.2±12.9			29.3±12.4				
2	33.4±11.7			32.5±10.9				
3	32.2±11.4			34.8±13.8				
4-5	33.6±12.5			33.5±10.7				
Metastatic LNs	2.5±4.7	7.4±9.4	<0.001	3.0±5.1		5.8±9.4		<0.001

### Oncologic outcomes

**Figure 2:**
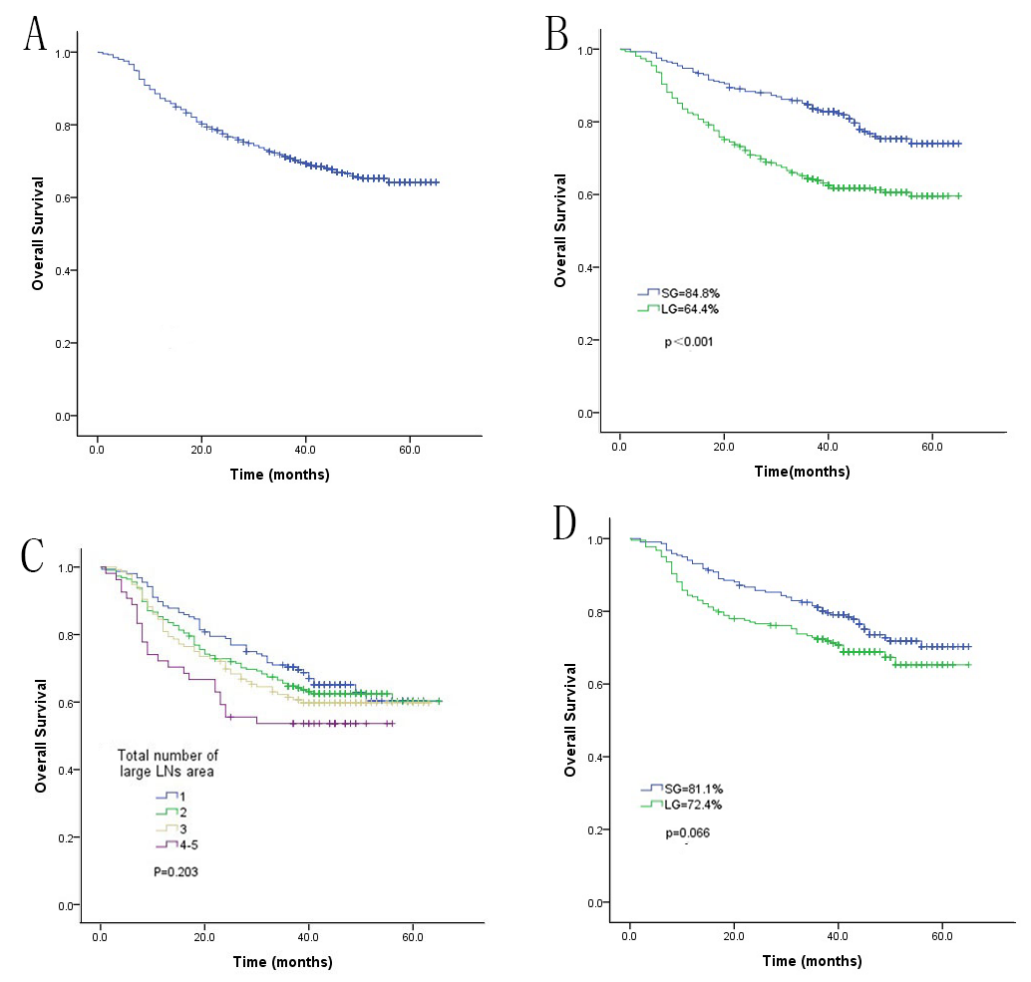
Comparison of the overall long-term survival rate between the LG and SG before and after matching **A**. Overall survival rate of all patients; **B**. comparison of the overall survival rate between the LG and SG before matching; **C**. comparison of the overall survival rate between patients with different total numbers of areas with enlarged LNs before matching; **D**. comparison of the overall survival rate between the LG and SG after matching.

Overall, 94.2% (835/855) of the patients completed a postoperative visit until the final follow-up date of June 2015, and the median follow-up period was 41 (range 3-65) months. The actual 3-year overall and recurrence-free survival rates of all patients were 71.2% and 67.3%, respectively. Before matching, patients in the LG had significantly lower 3-year overall and recurrence-free survival rates than patients in the SG (64.4% vs. 84.8% and 60.2% vs. 79.2%, respectively, *p* < 0.001). Additionally, in the LG, the 3-year overall survival rates were similar among patients with different total numbers of areas with enlarged LNs (Figure 2). After matching, the 3-year overall and recurrence-free survival rates of the LG were also comparable with those of the SG (81.1% vs. 72.4% and 76.4% vs. 67.5%, respectively, *p* > 0.05). Although the patients with stage IB or IIA GC tended to have a significantly different 3-year overall survival rates, additional Breslow and Tarone-Ware tests demonstrated that the 3-year overall survival rates were similar between the LG and SG when in stage IB (*p* = 0.121 and 0.123, respectively) or IIA (*p* = 0.124 and 0.120, respectively).

### Multivariate and stratified analyses of survival after matching

**Figure 3:**
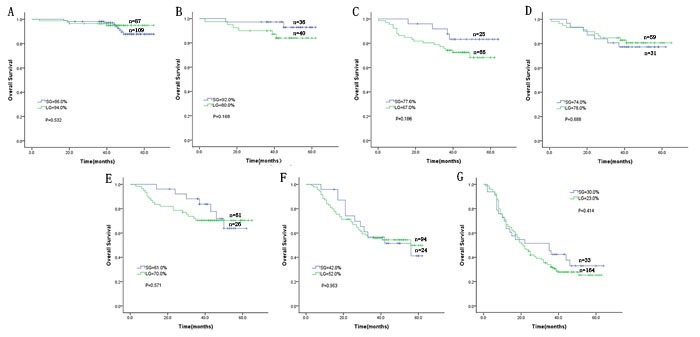
Comparison of the overall long-term survival rate between the LG and SG before matching according to stage **A**. Stage IA; **B**. stage IB; **C**. stage IIA; **D**. stage IIB; **E**. stage IIIA; **F**. stage IIIB; and **G**. stage IIIC.

**Figure 4:**
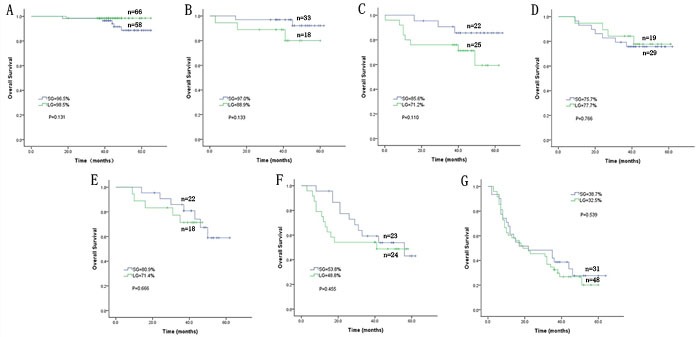
Comparison of the overall long-term survival rate between the LG and SG after matching according to stage **A**. stage IA; **B**. stage IB; **C**. stage IIA; **D**. stage IIB; **E**. stage IIIA; **F**. stage IIIB; and **G**. stage IIIC.

Univariate and multivariate Cox regression showed that pTNM stage was the only independent prognostic factor (Table 3). Both before and after matching, stratified analysis according to pTNM stage demonstrated that the 3-year overall survival rates at each stage were similar between the LG and SG (*p* > 0.05) (Figure 3, Figure 4).

**Table 3:** Cox Regression Analysis of Survival After Matching

Characteristic	Univariate				Multivariate			
	B	SE	Wald	p	B	SE	Wald	p
Gender	0.291	0.201	2.088	0.149	/	/	/	/
Age	0.356	0.181	3.877	0.049	0.183	0.202	0.820	0.356
With or without enlarged LNs	0.330	0.181	3.317	0.069	0.115	0.343	0.112	0.738
Charlson score	0.297	0.172	2.998	0.083	0.124	0.203	0.374	0.541
Lymphadenectomy	2.412	0.457	27.894	<0.001	0.390	0.568	0.471	0.492
Gastrectomy extent	0.807	0.170	22.497	<0.001	0.210	0.183	1.323	0.250
Tumor location	0.222	0.095	5.519	0.19	/	/	/	/
Tumor diameter	1.024	0.135	57.106	<0.001	0.266	0.173	2.364	0.124
Areas with enlarged LNs	0.138	0.071	3.762	0.052	0.031	0.140	0.048	0.826
Histologic type	0.010	0.210	0.002	0.961	/	/	/	/
pN	0.772	0.079	94.642	<0.001	0.164	0.242	0.460	0.498
pT	0.833	0.097	73.227	<0.001	0.153	0.230	0.440	0.507
pTNM	0.506	0.050	101.405	<0.001	0.565	0.214	6.972	0.008

## DISCUSSION

The incidence of GC is high in East Asia. In Japan and South Korea, early GCs account for more than 50% of all GCs. Since Kitano reported on the efficacy and advantages of LAG for early GC in 1994, LAG has gradually been adopted as the standard surgery for early GC. However, in China, most patients with GC are diagnosed at an advanced stage, often with enlarged perigastric LNs. The development of techniques, apparatuses and instruments has made it possible for LAG to be applied in advanced GC, with satisfactory surgical results. However, despite an abundance of knowledge, no studies have directly compared the oncologic results of LAG for GC with and without preoperative enlarged LNs. Thus, the impact of preoperative enlarged LNs on LAG outcomes remains unknown. Moreover, more often than not, patients with enlarged LNs present at a more advanced stage. In such cases, a simple case-control study may result in inaccuracies. Therefore, our study attempted to apply the propensity score-matching method to compare the long-term oncologic results of LAG in patients with and without preoperative enlarged LNs to determine the effect of perigastric LNs. Enlarged perigastric LNs complicate the anatomy by compressing or wrapping blood vessels or damaging the local fascia structure; this can result in inaccurate localization of the anatomical level or the dissection of blood vessels mistaken for enlarged LNs, thereby increasing the difficulty and risk of LN dissection. Therefore, the main concern of researchers is the curative effect of LN dissections, which is evaluated primarily based on the number of LNs dissected. The results of our study show that although the total number of LNs dissected was significantly greater in LG patients than in SG patients before matching, this sharp contrast disappeared after matching and pTNM stratification analysis. Moreover, the total number of LNs dissected did not decrease with an increase in the total number of areas with enlarged LNs. The amplification effect of the laparoscope could help surgeons to better identify the anatomical space between enlarged LNs and blood vessels and the surrounding structures by revealing more subtle vasculature, nerves and fascia. Moreover, the laparoscopic view provides the surgeon with a better view of the anatomical complexities when using an ultrasonic knife; this ensures more refined incisions, more effective hemostasis and less damage to the surrounding tissues, thus enabling the safe dissection of enlarged LNs at the root. Considering its ability to achieve high ligation of blood vessels and complete resection of LNs, it can be safely concluded that laparoscopic surgery has advantages in the dissection of enlarged LNs.

Long-term survival after surgery is an important aspect of LAG. In Japan and South Korea, the proportion of early GC patients receiving laparoscopic surgery has been growing rapidly. The long-term follow-up conducted in randomized controlled trials (RCTs) shows that LAG for early GC can achieve long-term efficacy equivalent to that of open surgery. Mochiki et al. [21] reported that they found no significant difference in the five-year survival rate between 89 cases of LAG and 60 cases of open surgery for early GC, with rates of 98% and 95%, respectively. The safety and long-term results of LAG for advanced GC have been confirmed by retrospective studies [22, 23]. Multi-center clinical trials regarding long-term oncologic results are underway as well. However, little attention has been paid to the long-term survival of patients with enlarged LNs undergoing LAG. Our data show that before matching, the 3-year overall survival rate of LG patients was significantly lower than that of SG patients, whereas after matching, no significant difference was observed. The 3-year overall survival rate was also similar among LG patients with a greater number of regions with enlarged LNs. Univariate and multivariate Cox regression showed that pTNM stage was the only independent prognostic factor. Before matching, patients in the LG had a significantly lower survival rate than those in the SG, which may have been due to a more advanced tumor stage in the LG. After matching, the LG and SG were in similar tumor stages. As a result, the groups had similar 3-year overall survival rates, and stratified analysis by each pTNM stage also demonstrated comparable 3-year overall survival between the two groups after matching. Therefore, for patients in the same tumor stage, having enlarged LNs does not affect the long-term efficacy of LAG, and the number of regions with enlarged LNs does not affect long-term survival either. Thus, LAG can be safely and effectively applied in GC patients with enlarged LNs.

This study not only had a long follow-up period but also employed a matched case-control design to remove the influence of confounding factors such as sex, age, extent of LN dissection, degree of tumor differentiation and pTNM. Thus, our results are reliable. However, our study still has certain limitations. In particular, being retrospective, this study may have inevitable hidden bias. Although this study adopted propensity score matching to balance the tumor staging and preoperative conditions between the two groups, the pN stages of patients in the LG were comparatively advanced, and fewer LG patients chose NC, which may have influenced the results to a certain extent. Although we used propensity score matching to increase the credibility of the retrospective study design, there was still possible selection bias, with inadequate variables (resection method and extent of lymphadenectomy) used to perform the propensity score matching. A decrease in bias would be important to determine the prognostic value of enlarged LNs in gastrectomy patients. Therefore, this surgical approach's exact long-term efficacy is yet to be confirmed and should be studied using a larger sample and a multi-center RCT.

Sponsored by the National Key Clinical Specialty Discipline Construction Program of China (No. [2012]649) and the Key Project of Science and Technology Plan of Fujian Province, China (Grant No. 2014Y0025).
